# A height-weight formula to measure body fat in childhood obesity

**DOI:** 10.1186/s13052-022-01285-8

**Published:** 2022-06-21

**Authors:** Maria Rosaria Licenziati, Giada Ballarin, Gabriella Iannuzzo, Maria Serena Lonardo, Olivia Di Vincenzo, Arcangelo Iannuzzi, Giuliana Valerio

**Affiliations:** 1 Department of Neurosciences, Obesity and Endocrine Disease Unit, Santobono-Pausilipon Children’s Hospital, Naples, Italy; 2 Department of Movement Sciences and Wellbeing, University of Naples “Parthenope”, Naples, Italy; 3 Department of Clinical Medicine and Surgery, Federico II University of Naples, Naples, Italy; 4 Department of Public Health, Federico II University of Naples, Naples, Italy; 5 Department of Medicine and Medical Specialties, A. Cardarelli Hospital, Naples, Italy

**Keywords:** Bioimpedance analysis, Body composition, Children, Height-weight equation, Obesity

## Abstract

**Background:**

The assessment of body composition is central in diagnosis and treatment of paediatric obesity, but a criterion method is not feasible in clinical practice. Even the use of bioelectrical impedance analysis (BIA) is limited in children. Body mass index (BMI) Z-score is frequently used as a proxy index of body composition, but it does not discriminate between fat mass and fat-free mass. We aimed to assess the extent to which fat mass and percentage of body fat estimated by a height-weight equation agreed with a BIA equation in youths with obesity from South Italy. Furthermore, we investigated the correlation between BMI Z-score and fat mass or percentage of body mass estimated by these two models.

**Methods:**

One-hundred-seventy-four youths with obesity (52.3% males, mean age 10.8 ± 1.9) were enrolled in this cross-sectional study. Fat mass and percentage of body fat were calculated according to a height-weight based prediction model and to a BIA prediction model.

**Results:**

According to Bland–Altman statistics, mean differences were relatively small for both fat mass (+ 0.65 kg) and percentage of body fat (+ 1.27%) with an overestimation at lower mean values; the majority of values fell within the limits of agreement. BMI Z-score was significantly associated with both fat mass and percentage of body fat, regardless of the method, but the strength of correlation was higher when the height-weight equation was considered (*r* = 0.82; *p* < 0.001).

**Conclusions:**

This formula may serve as surrogate for body fat estimation when instrumental tools are not available. Dealing with changes of body fat instead of BMI Z-score may help children and parents to focus on diet for health.

**Supplementary Information:**

The online version contains supplementary material available at 10.1186/s13052-022-01285-8.

## Background

Obesity is defined as excessive fat accumulation that presents a risk to health. Epidemiological studies, using either direct or indirect body composition methods, suggested a positive association between fat mass and mortality in the general population [1]. Therefore, the assessment of body composition is central in diagnosis and treatment of obesity, yet there are several limitations to estimate body fat (BF), especially in children and adolescents.

Dual-energy X-ray absorptiometry (DXA) and air displacement pletismography are considered as criterion methods for body composition assessment in adults, but not in children. Another approach is the use of bioelectrical impedance analysis (BIA), which estimates total body water, fat-free mass (FFM) and fat mass (FM) through predictive equations based on different variables, such as age, stature and eventually weight [2, 3]. However, the use of BIA in pediatric clinical practice is limited because it requires a specialised staff, standard conditions and a relative collaboration of the patients [4]. Therefore, several methods have been developed to assess adiposity from equations based on anthropometric measurements, such as weight and height, which are practical, repeatable and less influenced by the operators [5]. In this direction, body mass index (BMI) has been recommended to evaluate overweight and obesity in healthcare or clinical settings. Despite many strengths, BMI has some limitations because FM and FFM can differ among subjects with the same BMI [3]. Moreover, BMI is a sex and age related measurement, therefore BMI-for-age percentile or its equivalent (BMI Z-score) are the main tools used for the diagnosis of overweight and obesity [2].

BMI Z-score is frequently used as a proxy index of body composition also in follow-up studies [4]. However, it is of difficult interpretation for non-professionals, such as parents, and its meaning is not directly attributed to BF. In addition, BMI z-score is flattened at very high BMI values, leading to erroneous conclusions when considering variations over time [5–7]. BMI and BMI Z-score may not reflect changes in body composition in children and adolescents because body proportions and BF levels change during growth in a non-linear manner. For this reason, other anthropometric equations have been proposed to estimate FM%. For instance, some Authors tested different exponential numbers in the denominator of the weight/height formula [8]. In particular, the triponderal mass index (weight/height^3^) was more accurate than BMI for estimating BF levels in different pediatric populations [9, 10].

Recently, Hudda et al. developed a ‘height–weight equation’ to calculate FM in children and adolescents, which was validated against deuterium dilution in a large multi-ethnic dataset of UK children of different ethnic groups. [11]. This equation had a very high predictive ability, yielding a mean difference between observed and predicted fat mass of − 1.29 kg (95% confidence interval − 1.62 to − 0.96 kg). Furthermore, using the deuterium dilution method as a reference standard, this model provided estimates of FM as accurate as those yielded by DXA and BIA [12]. This equation considers also ethnicity as a predictor variable, distinguishing children of different ethnic origins. In a previous study, we found that FM estimated by the Hudda’s equation was independently associated with subclinical atherosclerosis in an Italian sample of children and adolescents with obesity [13].

Considering the interest toward a simple and feasible method to estimate BF, we aimed to assess the extent to which FM and percentage of BF (BF%) agreed by comparing two feasible methods to estimate BF, namely the height-weight equation by Hudda et al. and a BIA model in a sample of children and adolescents with obesity living in South Italy. As secondary outcome, we compared the correlation between BMI Z-score, and FM or BF% estimated by these two models.

## Methods

This retrospective study involved 174 Italian children and adolescents with obesity (52.3% males, mean age 10.8, range age 7–15 years) consecutively admitted for a first visit to the Obesity Unity, Santobono Pausilipon Children Hospital in Naples, Italy, from April 2017 to December 2017. To be included in the study the following criteria were considered: age 4–15 years, diagnosis of primary obesity, no previous weight loss treatment. Overweight, participation to competitive sports, assumption of medications that may affect body weight, or genetic, endocrine and iatrogenic causes of obesity were exclusion criteria. The institutional review board approved the clinical protocol and written informed consent for all procedures was obtained from all the children and/or their parents or legal guardians before the enrolment.

### Anthropometry, pubertal and lifestyle assessment

Anthropometric measurements were assessed by the same investigator, specifically trained. Standing height was determined by a Harpenden Stadiometer (Holtain Limited, Crymych, Dyfed, UK). Body weight was measured to the nearest 0.1 kg, by using standard equipment. BMI was calculated as weight divided for height^2^ (kg/m^2^). The WHO standards for age-and sex-specific BMI percentiles were used for calculating the BMI standard deviation score (SDS). Obesity was defined by BMI Z-score > 2 [14]. Pubertal stage was assessed by a paediatrician. Prepubertal stage was defined by Tanner Stage I of breast development in girls and testicular volume in boys [15].

Lifestyle was assessed by questionnaire and included information about participation in competitive or recreational sports in the previous 6 months (yes/not) and the weekly hours of training.

### Bioelectrical impedance analysis

BIA on the whole body (BIA 101 RJL, Akern, Florence Italy) was performed by the same investigator in standardized conditions: ambient temperature between 23–25 °C, fast > 3 h, empty bladder, and supine position for 10 min. Participants were asked to lie down with their legs and arms slightly abducted to ensure no contact between body segments. The measuring electrodes were placed on the anterior surface of the wrist and ankle, and the injecting electrodes on the dorsal surface of the hand and the foot, respectively [16].

### Body composition calculations

FM was calculated according to the height-and-weight-based prediction model, as suggested by Hudda et al. [11] and to the BIA prediction model, as suggested by Horlick et al. [17].

According to Hudda et al. [11] the following equation was used:

$$\mathrm{FM}\;(\mathrm{kg})\;=\;\mathrm{Weight}\;-\;\exp\;\lbrack0.3073\;\times\;\mathrm{height}^2\;-\;10.0155\;\times\;\mathrm{weight}^{-1}\;+\;0.004571\;\times\;\mathrm{weight}\;+\;0.01408\;\times\;\mathrm{BA}\;-\;0.06509\;\times\;\mathrm{SA}\;-\;0.02624\;\times\;\mathrm{AO}\;-\;0.01745\;\times\;\mathrm{other}\;-\;0.9180\;\times\;\ln(\mathrm{age})\;+\;0.6488\;\times\;\mathrm{age}^{0.5}\;+\;0.04723\;\times\;\mathrm{sex}\;+\;2.8055\rbrack$$

exp = exponential function; ln = natural logarithmic transformation; score 1 if child is of black (BA), south Asian (SA), other Asian (AO), or other (other) ethnic origins and score = 0 if not; sex = 1 for male and sex = 0 for female.

For the BIA estimation model, the following equation, previously validated in children aged 4–18 years by Horlick et al. [17], was used:

$$\mathrm{FM}-\mathrm{BIA}(\mathrm{kg})\;=\;\mathrm{weight}\;-\;\lbrack3.474\;+\;0.459\;\mathrm x\;\mathrm{height}^2\;/\mathrm R\;+\;0.064\;\mathrm x\;\mathrm{weight}\rbrack\;/\;\lbrack0.769\;-\;0.009\;\mathrm x\;\mathrm{age}\;-\;0.016\;\mathrm x\;\mathrm{sex}\rbrack$$

R = electrical resistance; sex = 1 for male and sex = 0 for female.

BF% was calculated for both equations as FM/weight × 100.

### Statistical analyses

Statistical Package for Social Sciences (version 26.0, SPSS, Inc., Chicago, Illinois) was used for statistical analyses. The Shapiro–Wilk test was performed to assess the normal distribution of the variables. Data not normally distributed were logarithmically transformed before analyses; for clarity of interpretation, results are expressed as untransformed values. Continuous variables were described as mean ± SD. Unpaired and paired student’s T-tests were used to compare means between boys and girls and within the same sex. Differences between the two estimation methods were normally distributed. Thus, Bland–Altman statistics [18] was used to compare the two models of FM and BF%. General linear model was performed to exclude any interactions between mean differences of the selected equations and gender or gender and age. Unadjusted and adjusted linear regression models were performed between the differences of the two methods and their corresponding averages. Partial correlation adjusted for gender, age and prepubertal stage was performed to compare the association between weekly hours of sports participation or BMI Z-score and FM or BF% estimated by the two different equations.

## Results

General characteristics of the study group as a whole or stratified by gender are shown in Table 1. No statistical differences were found for age, weight and stature, while BMI-Z score was significantly higher in boys than girls. BF% was significantly higher in girls than boys regardless the method used to assess the body composition, while there was no significant difference in FM. Only 37% of girls and 49% of boys participated in recreational sports. FM and BF% significantly differed between the two methods in girls, while no statistical differences were found in boys.

**Table 1:** Demographic, anthropometric and body composition data in the study sample considered as a whole or stratified by gender

	**Total (*n* = 174)**	**Boys (*n* = 91)**	**Girls (*n* = 83)**	p*
Age (yrs)	10.8 ± 1.9	10.9 ± 1.9	10.7 ± 1.9	0.558
Weight (kg)	70.2 ± 16.5	70.6 ± 16.5	69.9 ± 16.6	0.794
Prepubertal/pubertal %	43.5/56.5	54.2/45.8	33.3/66.7	0.011
Stature (cm)	148.4 ± 11.3	149.4 ± 11.3	147.2 ± 11.2	0.194
Body Mass Index (kg/m^2^)	31.5 ± 3.8	31.1 ± 3.4	31.8 ± 4.2	0.243
Body Mass Index Z-score	3.6 ± 0.7	3.7 ± 0.6	3.4 ± 0.7	0.004
Fat mass _height-weight equation_ (kg)	31.3 ± 8.1	30.2 ± 7.3	32.5 ± 8.8^a^	0.060
Fat mass _BIA_ (kg)	30.7 ± 9.0	30.0 ± 8.6	31.4 ± 9.5^b^	0.296
Body fat _height-weight equation_ (%)	44.4 ± 3.5	42.8 ± 2.6	46.3 ± 3.4^a^	< 0.001
Body fat _BIA_ (%)	43.2 ± 4.8	42.1 ± 4.3	44.4 ± 5.1^b^	0.002

a vs b = *p* < 0.001 within girls

^*^differences between genders

Partial correlation analysis did not show any significant relationship between weekly hours of sports and FM or %BF, adjusting for sex, age and prepubertal stage (FM _height-weight equation_ r -0.076, %BF _height-weight equation_ r – 0.156; FM_BIA_ r -0.042; %BF_BIA_ r -0.048).

**Fig. 1:**
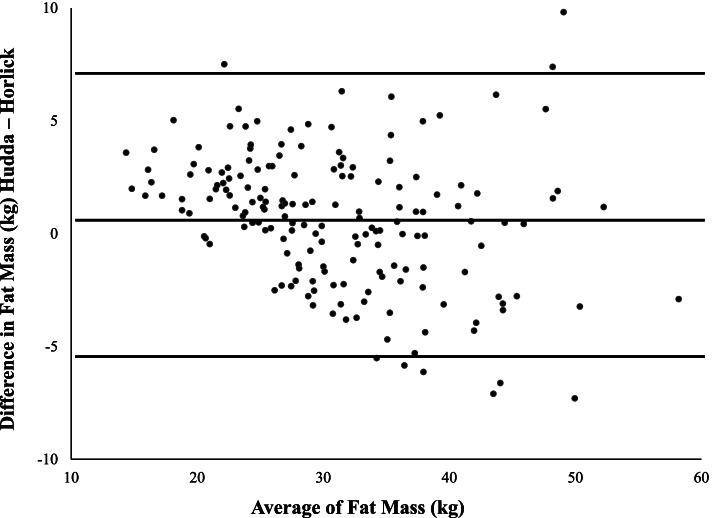
Comparison of height-weight or biompedance based equations for determining fat mass in the whole sample of youths with obesity

**Fig. 2:**
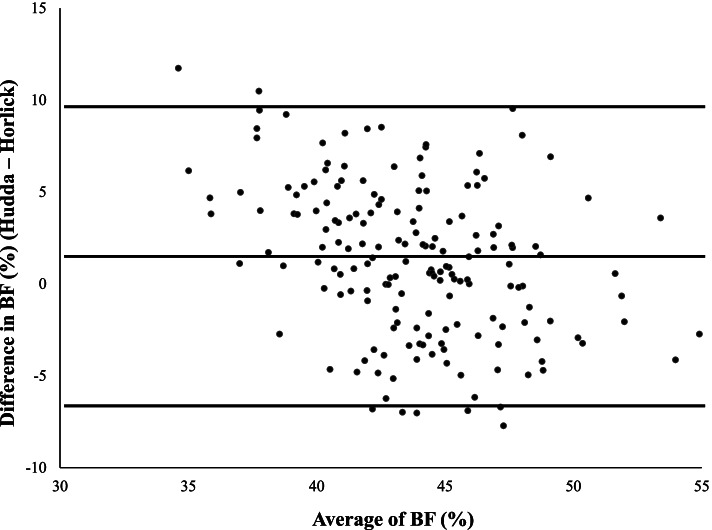
Comparison of height-weight or biompedance based equations for determining percentage of body fat in the whole sample of youths with obesity

Bland–Altman analysis was performed in the whole study group. The agreements of FM or BF% estimated by the two methods are shown as plots (Figs. 1 and 2), while statistics was synthetized in Table 2. The mean differences were relatively small for both FM and BF% and the majority of values fell within the upper and lower limits of agreement. Specifically, the averages of the differences in FM or BF% were positive and significantly different from 0 in the comparison of both equations (Table 2). Of note, a systematic error was more evident in BF% differences with an overestimation at lower mean values.

**Table 2:** Bland–Altman statistics, correlation and partial correlation in the study sample

	Mean difference	confidence intervals for 95% limits of agreement	R	r adjusted for gender, age and pubertal status
Δ FM	0.65*	-5.2; + 6.5	-0.310**	-0.174*
Δ BF%	1.27*	-6.8; + 9.4	-0.376**	-0.470**

*FM* Fat mass, *BF%* Percentage of body fat

Δ = differences between height-weight equation and bioimpedance equation

^*^p < 0.001 (mean differences vs. 0)

^**^p < 0.001

^*^p < 0.04

Compared to unadjusted coefficients of correlations, partial correlation analysis adjusted for gender, age and prepubertal stage showed a stronger negative correlation between differences and averages in both FM and BF% (Table 2).

Partial correlation adjusted by gender, age and prepubertal stage between BMI Z-score and FM or BF% estimated by different equations is shown in Table 3. BMI Z-score was significantly associated both with absolute and relative FM distribution estimated with either method. However, the relationship was greatly higher when the height-weight equation was used.

**Table 3:** Partial correlation adjusted by gender, age and prepubertal stage in the study sample

	BMI Z-score	*P*
Fat mass _height-weight equation_ (kg)	0.837	< 0.001
Fat mass _BIA_ (kg)	0.718	< 0.001
Body fat _height-weight equation_ (%)	0.837	< 0.001
Body fat _BIA_ (%)	0.505	< 0.001

*BIA* Bioimpedance analysis

## Discussion

The present study found a fair agreement in the estimation of BF between a height-weight equation [11] and a validated model of body composition assessment based on BIA [17] in a sample of children and adolescents with obesity living in South Italy. As far as we know, few studies have compared anthropometric estimation of body composition to BIA equation [12], while a robust body of literature exists on the comparison of anthropometric or BIA equation with DXA or air displacement pletismography [19].

Recently, Hudda et al. proposed a ‘height–weight equation’ to estimate FM in children and adolescents, which was validated against to deuterium dilution [11]; this equation provided FM estimation at least as accurate as DXA and BIA [12]. In a retrospective study performed in a Danish cohort, FM estimated by this height-weight equation at 10 or 13 years was associated with adult risk of type 2 diabetes between 30 and 70 years better than childhood weight [20]. Furthermore, FM estimated by this equation was independently associated with subclinical atherosclerosis [13] and showed a good ability for detection of metabolic syndrome in Italian children and adolescents [21].

The present study provides further insights by comparing the extent to which FM and BF% estimated by the Hudda’s prediction agreed with another feasible model based on BIA in a clinical sample of Italian youths with obesity. Both equations revealed the expected gender differences in body composition. However, the height-weight equation provided slightly higher values of FM and BF% compared to the BIA method in girls, while no significant differences were found in boys. By using the Bland–Altman analysis we found a difference between the two methods of only 0.65 kg in the estimation of FM and 1.27% in the estimation of BF%. Considering as ideal an agreement of zero difference between measurements, the comparison between the two methods is acceptable. Yet, the height-weight equation overestimated the amount of FM or BF% at lower mean values compared to the BIA equation, even after controlling by gender, age and prepubertal stage. Since measures of BF in children are dependent on individual characteristics (such as age, height, gender and pubertal status), differences are expected when body composition is estimated with different methods. Indeed, errors in estimation of FM using BIA and DXA were demonstrated by Reilly et al. [22] in a sample of normal weight children, and by Lazzer et al. [23] in a sample of youths with severe obesity. Furthermore, Hudda et al. [12] demonstrated that the accuracy of DXA and BIA assessments in paediatric age varied considerably in children across the range of FM values, with DXA overestimating FM at higher levels and underestimating at lower levels.

As secondary outcome, we found a greater relationship between BMI Z-score and FM or BF% as estimated by the height–weight equation than the BIA prediction model, after adjusting for gender, age and prepubertal stage. As far as we know, only Calcaterra et al. have assessed the correlation between the height-weight equation with other indices of obesity, such as BMI (*r* = 0.69, *p* < 0.001), waist circumference (*r* = 0.32, *p* < 0.01) [21].

A recent systematic review reported that BMI Z-score was more frequently used than BMI or BMI percentage (24%, 9% and 13%, respectively) to assess weight loss in paediatric obesity; DXA or BIA were by far under-reported (5% and 1% respectively) [4]. The BMI Z-score is a measure of relative weight adjusted for child age and sex, used to classify overweight and obesity and to monitor weight loss, but the information associated to this index is difficult to explain to parents and children. A practical application of the height-weight equation compared to BMI Z-score is shown in the Supplementary table. Two examples are presented, showing either 3.0 kg of weight increase or 1.0 kg of weight loss after 6 months of treatment in a 10.5 year-old boy with a linear growth of + 3 cm. In both cases a reduction of BMI Z-score above 0.25 was hypothesized, an amount that has been deemed to be effective in reducing cardiovascular risk factors in obese children [24]. Reduction of 0.7 or 2.4 BF% was estimated according to the height-weight formula against to a reduction of BMI Z-score of 0.26 or 0.54, respectively.

Our study has several limitations. Firstly, the study group consisted of a small sample of children and adolescents with obesity, hence it is not representative of the general pediatric population. In addition, our results are applicable only to a Caucasian population. Secondly, we could not validate the formula against a reference method, such as DXA or deuterium dilution. On the contrary, one strength of our study is the use of two equations that were previously validated in youths with the same age range of our population against reference methods. Lastly, either the anthropometric variables or the BIA data were estimated by a unique operator, reducing the observer bias.

## Conclusions

We found a fair agreement between two simple-to-use tools to assess body composition in a sample of youths with obesity living in South Italy. Therefore, the height-weight formula may serve as surrogate for FM estimation when feasible instrumental tools, such as BIA are not available. Calculation of FM from the relevant predictor variables is simple, using the Excel calculator provided by the Authors [11]. The strict correlation between BMI Z-score and BF% estimated by the height-weight equation suggests that the two formulas may be interchangeable. The usefulness of the height-weight formula may rely on the opportunity to deal with changes of BF instead of weight or BMI Z-score, helping children and parents to focus on diet and exercise for health. Further studies are needed to compare the height-weight equation and BMI Z-Score against DXA in the estimation of BF in children. Whether the height-weight formula should perform better than BMI, the development of adiposity-related cut offs specific for gender and age, might help to better define pediatric obesity.

## Supplementary Information

**Additional file 1: Supplementary table 1.** Changes of BMIZ-score or body fat percentage estimated by the height-weight equation in anadolescent boy after 6 months of treatment. Example: comparison between changesin BMI Z-score and body fat percentage in an adolescent boy after 6 months of treatment.

## Data Availability

The datasets used and/or analysed during the current study are available from the corresponding author on reasonable request.

## Abbreviations

BIA: Bioelectrical impedance analysis
BF: Body fat
BMI: Body mass index
DXA: Dual-energy X-ray absorptiometry
FFM: Fat-free mass
FM: Fat mass
