# Supplementary material for: A height-weight formula to measure body fat in childhood obesity
Source: Ital J Pediatr. 2022 Jun 21;48:106. doi: 10.1186/s13052-022-01285-8 (PMC9210685; doi:10.1186/s13052-022-01285-8)
Supplement: Supplementary file 1 — Additional file 1: Supplementary table 1. Changes of BMIZ-score or body fat percentage estimated by the height-weight equation in anadolescent boy after 6 months of treatment. Example: comparison between changesin BMI Z-score and body fat percentage in an adolescent boy after 6 months of treatment. [file 13052_2022_1285_MOESM1_ESM.docx]

Supplementary table 1. Changes of BMI Z-score or BF% estimated by the height-weight equation in an adolescent male after 6 months of treatment.

|  | T0 | T6 (a) | Changes | T6 (b) | changes |
| --- | --- | --- | --- | --- | --- |
| Age (years) | 10.5 | 11.0 | 0.5 | 11.0 | 0.5 |
| Weight (kg) | 73.0 | 76.0 | 3.0 | 72.0 | 1.0 |
| Stature (m) | 1.52 | 1.55 | 0.03 | 1.55 | 0.03 |
| BMI (kg/m^2^ ) | 31.6 | 31.6 | 0 | 30.0 | -1.6 |
| BMI Z-score | 3.89 | 3.63 | -0.26 | 3.35 | -0.54 |
| Body fat (%) | 44.4 | 43.7 | -0.7 | 42.0 | -2.4 |

T6 (a) weight increase after 6 months; T6 (b) weight loss after 6 months.
